# Supplementary material for: Predicting visual function by interpreting a neuronal wiring diagram
Source: Nature. 2024 Oct 2;634(8032):113–23. doi: 10.1038/s41586-024-07953-5 (PMC11446822; doi:10.1038/s41586-024-07953-5)
Supplement: Supplementary file 6 — CRF and ERF predictions for individual TmY4 and TmY9 cells. Analogous to Supplementary Data 3, but for TmY target types. Shown are the top four monosynaptic pathways, the strongest pathway passing through each of the top ten intermediary types (ranking from Extended Data Fig. 7), and the trisynaptic pathway Tm1–TmY–Dm3–TmY (see the section entitled Prediction of spatial normalization). [file 41586_2024_7953_MOESM6_ESM.zip › DataS4/TmY9q/720575940614057903.pdf]

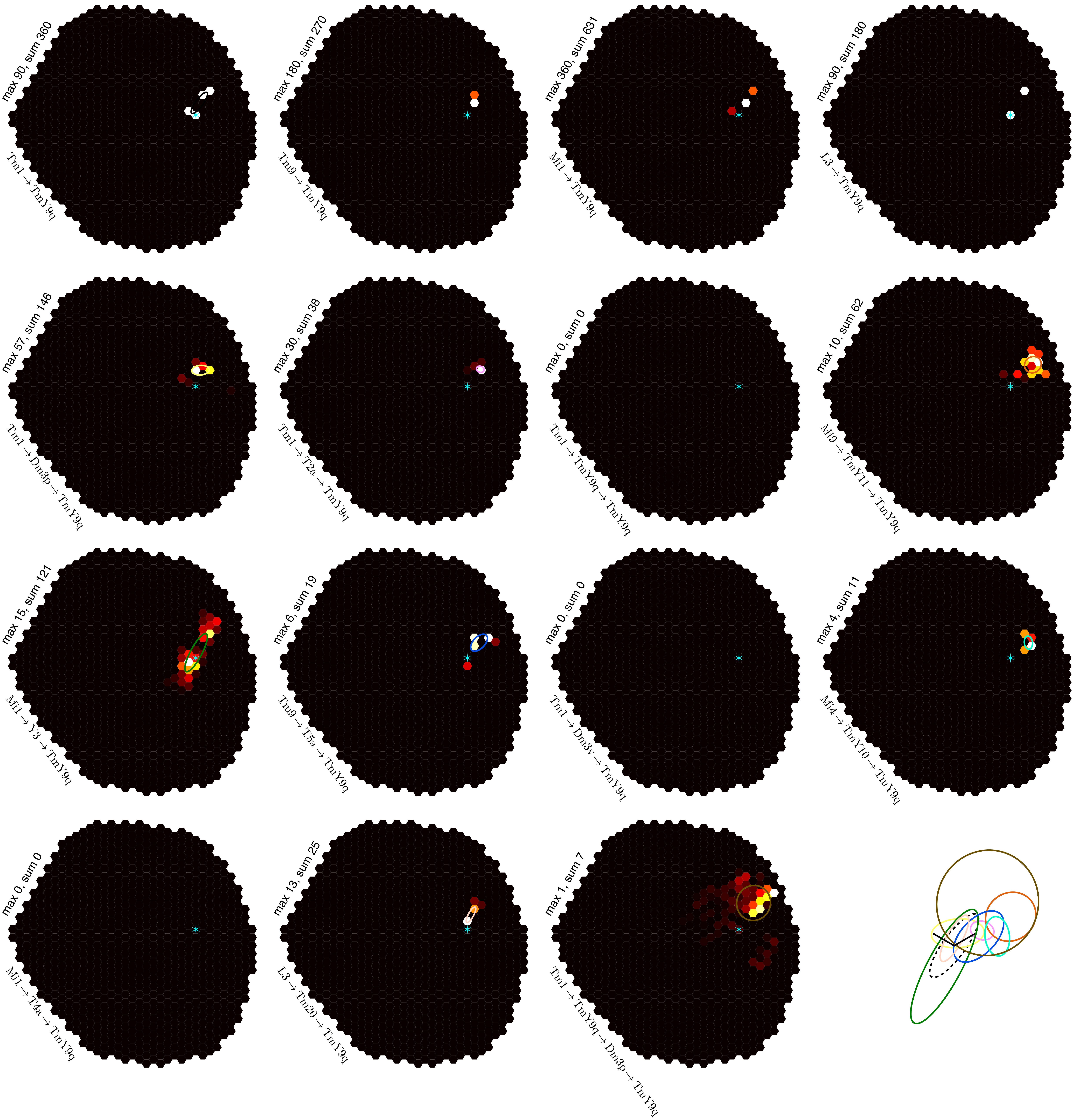
max 90, sum 360
Tm1 → TmY9q
max 180, sum 270
Tm9 → TmY9q
max 360, sum 631
Mi1 → TmY9q
max 90, sum 180
L3 → TmY9q
max 57, sum 146
Tm1 → Dm3p → TmY9q
max 30, sum 38
Tm1 → T2a → TmY9q
max 0, sum 0
Tm1 → TmY9q → TmY9q
max 10, sum 62
Mi9 → TmY11 → TmY9q
max 15, sum 121
Mi1 → Y3 → TmY9q
max 6, sum 19
Tm9 → T5a → TmY9q
max 0, sum 0
Tm1 → Dm3v → TmY9q
max 4, sum 11
Mi4 → TmY10 → TmY9q
max 0, sum 0
Mi1 → T4a → TmY9q
max 13, sum 25
L3 → Tm20 → TmY9q
max 1, sum 7
Tm1 → TmY9q → Dm3p → TmY9q
